# Lactate Metabolism in the Intervertebral Disc: Mechanistic Insights and Pathological Implications

**DOI:** 10.3390/biom16010170

**Published:** 2026-01-20

**Authors:** Ting Zhang, Peng Feng, Peter G. Alexander, Joon Y. Lee, Gwendolyn A. Sowa, Nam V. Vo

**Affiliations:** 1 Department of Orthopaedic Surgery, University of Pittsburgh, Pittsburgh, PA 15213, USA; 2 Department of Physical Medicine and Rehabilitation, University of Pittsburgh School of Medicine, Pittsburgh, PA 15219, USA

**Keywords:** intervertebral disc degeneration, lactate metabolism, histone lactylation, epigenetics regulation, metabolic symbiosis

## Abstract

The intervertebral disc (IVD) is the largest avascular structure in the human body, and its nucleus pulposus (NP) cells predominantly generate large amounts of lactate through glycolysis, accompanied by an acidic microenvironment—features that represent characteristic metabolic traits of disc cells. In recent years, knowledge of the biological roles of lactate has undergone a conceptual shift. On the one hand, lactate can serve as a context-dependent auxiliary biofuel in specific regions of the IVD, particularly within annulus fibrosus (AF) regions adjacent to the NP. On the other hand, lactate functions in disc cells as a signaling molecule and a metabolic–epigenetic regulator, influencing transcriptional programs through lactylation and modulating multiple molecular pathways associated with cellular stress adaptation and fate determination. This review summarizes current knowledge on lactate production, transport, and clearance in the intervertebral disc, as well as emerging evidence for the roles of lactate in disc health and pathophysiology. In addition, we outline research perspectives and future directions aimed at advancing our understanding of lactate biology and evaluating its potential as a therapeutic target for intervertebral disc degeneration.

## 1. Introduction

Low back pain (LBP) ranks among the leading causes of global disability [1], with intervertebral disc degeneration (IDD) recognized as a principal pathological driver [2]. Cells in the IVD, the largest avascular structure in the human body [3,4], exist in a microenvironment characterized by hypoxia, restricted nutrient supply, acidity, and mechanical strain. NP cells rely predominantly on anaerobic glycolysis for energy production, resulting in substantial lactate generation accompanied by an increased proton load, which contributes to acidification of the intervertebral disc microenvironment [5]. Under physiological conditions, lactate and protons produced by disc cells are exported across the plasma membrane via monocarboxylate transporters (MCTs) [6,7,8]. At the tissue level, small solutes and metabolic byproducts are primarily cleared through passive diffusion across the cartilage endplates (CEP) and the extracellular matrix of the intervertebral disc toward the adjacent blood supply [6,7,8]. This transport process is essential for maintaining intracellular pH homeostasis and metabolic balance in disc cells.

In recent years, accumulating evidence has revealed increasingly diverse roles of lactate. Isotope-tracing studies demonstrate that lactate derived from NP cells can be taken up by neighboring AF cells and utilized in cellular energy metabolism [9]. In addition, lactate serves as a substrate for lysine lactylation, contributing to post-translational modification of both histone and non-histone proteins, thereby regulating epigenetic states as well as enzymatic activity and signal transduction [5]. Given the unique lactate-rich metabolic environment of the intervertebral disc, elucidating the multifaceted roles of lactate is critically important for understanding disc pathophysiology.

This review systematically summarizes lactate production, transport, and clearance within the intervertebral disc, as well as lactate-driven metabolic coupling among distinct disc tissue compartments. We synthesize current evidence regarding the overall impact of lactate on disc homeostasis and degeneration, and its regulatory roles in signaling pathways and epigenetic modulation across different disc cell populations. Finally, we discuss key limitations of existing experimental models and unresolved questions, outline future research directions, and evaluate the translational potential of targeting the lactate metabolism–epigenetic network as a therapeutic strategy for intervertebral disc degeneration.

## 2. IVD Lactate Production

**Disc Structure and Nutrient Supply Mechanisms.** IVD is located between adjacent vertebral bodies and consists of a central NP, a surrounding AF, and CEPs on the superior and inferior sides. Cellular phenotypes and extracellular matrix composition undergo dynamic changes with disc maturation and health status, with disc homeostasis depending on the balance between matrix synthesis and degradation, a balance that is disrupted during degeneration. Significant interspecies differences exist in disc size, endplate structure, and cellular phenotypes, particularly in the transition of NP cells from a notochordal-like to a mature phenotype [10,11,12,13].

As the largest avascular structure in the human body, nutrient supply to disc cells relies primarily on passive diffusion driven by metabolic activity-induced concentration gradients. Cyclic spinal loading during daily activities facilitates fluid exchange in and out of the disc, thereby assisting nutrient transport. However, extensive experimental and theoretical evidence indicates that small molecules such as glucose and oxygen enter the disc predominantly by diffusion [14,15,16]. This restricted nutrient supply is essential for maintaining cell viability and metabolic activity, particularly within the central NP region [17,18,19,20,21,22,23,24,25,26].

**Production of IVD lactate by anaerobic glycolysis.** Owing to the absence of vascular supply, the IVD exists in a chronically hypoxic microenvironment, particularly within the central NP, where oxygen tension is lowest (approximately 5–15 mmHg) and lactate concentrations are highest (approximately 2–6 mM), thereby establishing a pronounced spatial metabolic gradient across the disc [18,27]. Under these conditions, disc cells rely almost exclusively on anaerobic glycolysis for energy production, in which pyruvate derived from glucose metabolism is reduced to lactate by lactate dehydrogenase A (LDH-A/LDH5) [9,28,29]. This process generates two molecules of lactate per molecule of glucose and is coupled with the regeneration of NAD^+^ from NADH, thereby sustaining glycolytic flux under anaerobic conditions [30,31,32,33].

Lactate levels within the IVD exhibit a consistent regional distribution, with the highest concentrations detected in the NP and a progressive decline toward the AF [7]. In human discs, NP lactate concentrations typically range from 2–6 mM, whereas levels in the outer AF are approximately 1 mM and can increase to 12–16 mM in severely degenerated discs [18]. Similar spatial gradients have also been reported in canine IVDs [30].

To adapt to the persistently hypoxic and nutrient-restricted microenvironment, disc cells undergo metabolic reprogramming orchestrated by hypoxia-inducible factor-1α (HIF-1α). HIF-1α enhances glycolytic flux by upregulating glucose transporters (GLUT1/3) and key glycolytic enzymes, while simultaneously inducing LDHA and activating pyruvate dehydrogenase kinase-1 (PDK1), thereby limiting pyruvate entry into oxidative phosphorylation and reinforcing lactate production [34,35,36,37]. In addition to glucose availability, lactate generation is tightly regulated by oxygen tension and extracellular pH; lactate-induced acidification feeds back to suppress further lactate synthesis, whereas increased oxygen availability reduces lactate yield, together forming a negative feedback loop governing lactate production in the disc [32,38].

## 3. IVD Lactate Transport, Accumulation, and Clearance

**MCT–Mediated Lactate Transmembrane Transport.** NP lactic acid must be exported to avoid intracellular accumulation and over acidification. Lactate transport across the cell membrane occurs through three routes: minimal passive diffusion of undissociated lactate, anion exchange systems, and—most importantly—proton-coupled transport mediated by MCTs [39]. MCTs constitute a family of proton-linked carriers that mediate bidirectional lactate flux according to lactate/H^+^ gradients. This process is ATP-independent and relies on passive co-diffusion [40,41]. MCT transport of lactate is coupled with protons (H^+^ ions), i.e., lactate enters or exits the cell with a proton with it, which helps maintain the overall charge balance across the cell membrane. Under hypoxia, MCT activity is essential for lactate transport, maintaining intracellular acid–base balance, and sustaining metabolic stability [42]. Within the hypoxic IVD, MCT1 and MCT4 are the predominant isoforms [43,44,45,46]. Their distribution is region-specific and complementary: MCT4 mediates lactate efflux, whereas MCT1 facilitates lactate influx into cells, thereby sustaining lactate gradients and supporting metabolic equilibrium within disc cells. CD147 (basigin/EMMPRIN) acts as a molecular chaperone required for proper localization and stability of MCT [47,48,49]. However, most insights into MCT–CD147 cooperation are derived from cancer biology [47,50,51,52], and whether a similar interaction exists in IVD cells remains to be elucidated.

**MCT4-mediated lactate export in IVD.** MCT4, encoded by the solute carrier family 16-member 3 (SLC16A3) gene, is a low-affinity, high-capacity lactate transporter highly expressed in glycolytic tissues [53,54]. Given the glycolytic dependence of NP cells, large amounts of lactate must be exported via MCT4 through a proton-coupled mechanism [55]. Under hypoxia, HIF-1α enhances MCT4 transcription by binding and activating an intronic enhancer within SLC16A3, while simultaneously suppressing mitochondrial respiration, promoting glycolytic flux, and facilitating lactate–proton co-export to maintain intracellular acid–base homeostasis [56,57,58]. In healthy IVDs, lactate released by NP cells via MCT4 diffuses across the cartilage endplate and is cleared by vertebral capillaries [14,17].

The essential role of MCT4 in disc homeostasis has been demonstrated in vivo and in vitro. Silagi et al. [6] showed that MCT4-deficient mice developed age-dependent IDD characterized by disrupted NP structure, reduced glycosaminoglycan (GAG) content, elevated matrix metalloproteinase-13 (MMP13), deterioration of vertebral trabeculae, and reduced bone quality. Metabolomic profiling and [^13^C]-glucose tracing further revealed that short-term inhibition of MCT4 under hypoxia caused intracellular lactate and proton accumulation, reduced extracellular acidification rate (ECAR), and increased pyruvate and tricarboxylic acid (TCA) intermediates, indicating a metabolic shift from glycolysis toward oxidative metabolism primarily via pyruvate dehydrogenase (PDH) activity [6]. Consistent with the glycolytic profile of NP cells, Wang et al. confirmed that MCT4 expression is markedly higher in NP than AF tissue [9], supporting the role of MCT4 in exporting lactate out of NP cells to maintain physiological pH.

**MCT1-mediated lactate import in IVDs.** MCT1 is a bidirectional, proton-coupled lactate transporter whose expression is upregulated by the transcription factor cellular myelocytomatosis oncogene (c-Myc) [41,59]. It is widely expressed in diverse cell types, with the direction of lactate flux determined by transmembrane gradients of lactate and H^+^. When extracellular lactate concentrations exceed intracellular levels, MCT1 facilitates cellular lactate influx. In IVDs, MCT1 is expressed highly in AF but not in NP tissue [9]. Upon exposure to exogenous lactate, AF cells further upregulate MCT1 expression, accompanied by increased lactate uptake and enhanced metabolic activity. ^14^C-lactate tracing experiments confirmed that AF cells take up lactate in a concentration-dependent manner, reinforcing the key role of MCT1 in AF cell lactate uptake [9]. Wang et al. [60] developed a five-layer three-dimensional (3D) NP degeneration model using gelatin sponges to mimic the progressively hypoxic and high-lactate microenvironment of the degenerating disc. In this model, inhibition of MCT1-mediated lactate influx by the specific inhibitor AZD3965 reversed the deleterious effects of lactate by restoring GAG accumulation, downregulating matrix metalloproteinase-3 (MMP3) expression, and alleviating NP cell degeneration. These findings suggest that blocking MCT1-dependent lactate uptake by NP cells can mitigate disc degeneration by reducing NP intracellular acid stress and catabolic activity [60]. Hence, lactate export out of NP cell is beneficial while lactate import into NP cells is harmful for IVD health.

**Mechanisms of lactate accumulation and its regulation.** Excessive lactate accumulation in the IVD reflects an imbalance in production, transport, and or clearance of lactate [6,61,62,63]. Structurally, the CEP is the principal egress route: age or degeneration-related calcification and sclerosis of the CEP that reduces permeability, impairs lactate clearance, and promotes IVD acidification [22,64,65]. CEP Modic changes and Schmorl’s nodes further compromise solute exchange [25,66]. Experiments have demonstrated that lactate diffuses more readily across the CEP than glucose, yet less efficiently than within AF or NP tissues [67]. These findings suggest that the CEP functions as a partial diffusion barrier [67]. Moreover, lactate diffusivity across the CEP correlates strongly with its porosity, underscoring the critical role of CEP microstructural integrity in regulating metabolite transport [67]. Finite element modeling further suggests that impaired CEP permeability disrupts disc nutrient homeostasis, exacerbating the imbalance between lactate accumulation and oxygen/glucose supply within the disc [38]. The cartilage endplate therefore represents the primary functional barrier controlling metabolite exchange between the disc and adjacent vertebral bodies [68].

External mechanical loading can modulate the metabolic microenvironment of the IVD, thereby partially alleviating lactate accumulation. Dynamic compression enhances solute convection and diffusion within the tissue, improving oxygen transport and promoting lactate clearance, as suggested by computational modeling studies [69]. Specifically, cyclic loading induces alternating tissue deformation, which facilitates solute transport and may elevate local oxygen availability within the disc. As oxygen availability and solute transport improve, the extracellular microenvironment may become less acidic. Such changes can influence cellular metabolic regulation and contribute to the establishment of a new metabolic steady state. Importantly, however, increased oxygen tension does not necessarily suppress glycolytic activity in disc cells, as cartilage-like tissues have been shown to exhibit a negative Pasteur effect, whereby glycolytic flux and lactate production may be maintained or even enhanced under higher oxygen availability [31]. Therefore, the beneficial effects of mechanical loading on lactate levels are more likely attributable to improved solute transport and clearance rather than direct suppression of glycolysis [69]. Computational modeling further supports this mechanism [62]. Simulations revealed that maintaining a state of moderate mechanical–transport coupling—where tissue deformation and porosity changes occur under sustained compressive loading—significantly improves oxygen transport within the IVD and reduces lactate accumulation. Even under continuous compressive loading, lactate levels were lower when mechanical loading was coupled with solute transport, thereby optimizing the disc’s metabolic and nutritional microenvironment [62]. Collectively, these findings suggest that refinement of mechanical loading strategies could represent a promising strategy to mitigate lactate buildup in degenerative disc conditions.

MCT greatly contributes to intracellular lactate accumulation and clearance from the cell. Beyond CEP permeability, steady-state lactate in the disc depends on MCT4-driven efflux from NP and MCT1-mediated uptake by AF/EP. Insufficient MCT4 elevates intracellular lactate/H^+^ and limits tissue-level clearance; inadequate or spatially mismatched MCT1 reduces downstream “sinks,” curbing redistribution to oxidative/signaling compartments. Thus, the balance of lactate export by MCT4 (NP) and import by MCT1 (AF/EP)—with proper membrane localization (e.g., CD147)—sets net lactate fluxes. In degenerative setting (e.g., CEP sclerosis, chronic hypoxia), even modest shifts favor lactate retention and microenvironmental acidification. Impaired lactate export and glycolytic dysregulation can promote lactate buildup and metabolic stress in NP cells. NP cells are adapted to the hypoxic disc environment, relying predominantly on glycolysis regulated by HIF-1α and its downstream targets such as PDK1 and LDHA [70,71]. When lactate efflux is inhibited, intracellular lactate and intermediates like glucose-6-phosphate (G6P) accumulate, consistent with feedback inhibition of glycolytic enzymes and subsequent acidosis [6].

**Acidic stress and cellular consequences of lactate accumulation.** Healthy NP has a pH of about 6.8. Thus, given the pKa of 3.9 of lactic acid, any lactic acid molecules produced in the hypoxic NP are readily dissociated into lactate and H^+^ which contribute to the acidity of the disc microenvironment [9]. Indeed, our study demonstrated that AF cells can tolerate relatively high levels of lactate, showing no adverse effects on cell viability or morphology up to 10 mM lactate, with only a modest reduction in viability observed at 20 mM [9]. But its lactic acid counterpart is harmful to IVD cells due to its ability to acidify the disc microenvironment. In the IVD, sustained accumulation of lactic acid creates an acidic microenvironment that activates acid-sensing ion channels (ASICs) [72]. This acidic milieu induces inflammatory responses and enhances catabolic activity in disc cells [72].

Evidence indicates that acidic stress not only impairs the physiological functions of NP cells but also upregulates pro-inflammatory cytokines and matrix-degrading enzymes, thereby accelerating ECM degradation and promoting the onset and progression of IDD [73]. Exposure of NP cell cultures to acidic stress (pH 6.5) for seven days upregulates mRNA levels of IL-1β and IL-6 by approximately 81-fold and 7.8-fold, respectively. Expression of neurotrophic factors such as nerve growth factor and brain-derived neurotrophic factor also increase significantly (3.0-fold and 4.6-fold, respectively). These changes are accompanied by activation of the NF-κB signaling pathway and elevated expression of matrix metalloproteinase-2 (MMP2) and matrix metalloproteinase-9 (MMP9), indicating that an acidic environment simultaneously drives inflammatory and catabolic cascades [74,75]. Additional in vitro studies have confirmed that acidic conditions suppress proteoglycan synthesis, upregulate IL-1β expression, and induce metabolic dysfunction and programmed cell injury in NP cells [76,77]. Collectively, these findings demonstrate that low extracellular pH—resulting directly from lactate accumulation—serves as a critical trigger for inflammatory activation and catabolic imbalance within the disc microenvironment.

Zhan et al. [78] further demonstrated that inflammatory mediators together with an acidic microenvironment resulting from metabolic activity in degenerative CEP cells can promote NP cell inflammation via a paracrine mechanism, thereby establishing an acid–inflammation signaling axis between the CEP and NP. To counteract this interaction, they developed a functional hydrogel system (CAP-sEXOs@Gel) composed of endplate-targeting engineered exosomes and a calcium carbonate/chitosan composite. This hydrogel alleviated acidic stress and inflammatory responses in both CEP and NP cells, and effectively delayed disc degeneration in animal models [78].

## 4. Emerging Roles of Lactate Metabolism and Inter-Tissue Coupling in the IVD

The AF and CEP regions adjacent to the NP exhibit transitional features in both cellular composition and extracellular matrix organization [68,79]. Recent studies have further identified a transition zone between the NP and AF, characterized by gradual changes in collagen fiber orientation and mechanical properties, and structurally integrated with neighboring tissues through anchorage, fiber penetration, or interweaving [79]. In parallel, the NP and CEP are anatomically and functionally highly continuous; as the principal interface linking the NP to vertebral blood supply, the CEP not only provides mechanical support but also regulates the diffusion of nutrients and metabolic byproducts, as well as associated signaling exchanges, thereby critically shaping the metabolic microenvironment and cellular homeostasis of the NP [68]. Collectively, this continuous yet regionally specialized structural architecture provides the physical basis for functional and metabolic interactions among the NP, AF, and CEP.

**Lactate as a Biofuel: Metabolic Symbiosis Between NP and AF.** Recent studies have revealed a lactate-mediated metabolic symbiosis within the IVD. Wang et al. [9]. were the first to employ heavy isotope ^13^C-labeled lactate to trace its metabolic fate in AF cells, demonstrating that lactate is converted into TCA cycle intermediates—including α-ketoglutarate, succinate, and malate—and incorporated into the biosynthesis of amino acids such as alanine and glutamate.

**Figure 1:**
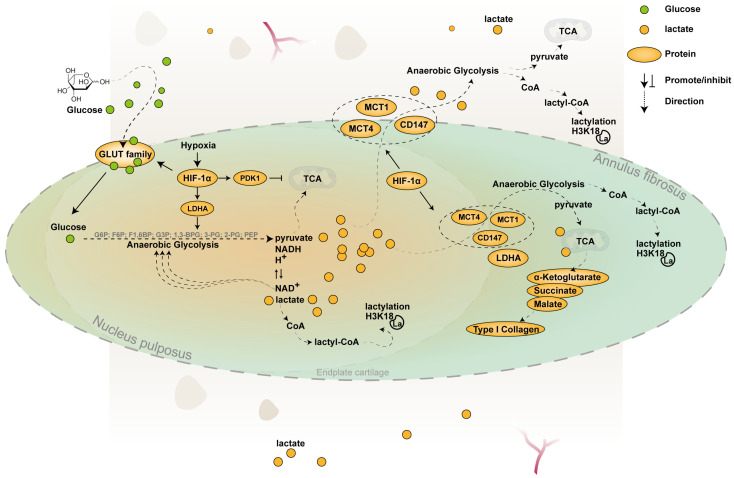
Pathways of lactate metabolism within the IVD. This schematic illustrates lactate production and transport within the intervertebral disc. In the hypoxic NP, HIF-1α is stably expressed and serves as a key transcription factor regulating glycolytic pathways [37]. It upregulates GLUT1 and GLUT3 and rate-limiting glycolytic enzymes such as hexokinase, phosphofructokinase, and pyruvate kinase, thereby enhancing glycolytic flux [35]. HIF-1α also promotes the expression of LDHA, facilitating lactate production [36], and induces PDK1, which limits pyruvate entry into mitochondrial oxidative pathways and favors anaerobic metabolism [37]. MCT1 and MCT4, two major monocarboxylate transporters, interact with the molecular chaperone CD147, which regulates their membrane localization and stability [47,48]. Under hypoxia, HIF-1α also upregulates MCT4 expression by activating an intronic enhancer within the *SLC16A3* gene [56,57]. Lactate diffuses toward the AF, where it can be imported via MCT1 and utilized for energy metabolism and matrix synthesis, as illustrated in the figure [9]. EPs rely on MCT1 to import lactate derived from NP cells, using it both for energy metabolism and histone H3K18 lactylation. This epigenetic modification contributes to the trans-differentiation of EP cells from cartilage toward subchondral bone [80].

Functionally, lactate exposure increased the mitochondrial oxygen consumption rate of AF cells by approximately 50% and upregulated type I collagen synthesis, indicating efficient utilization of lactate for both energy production and matrix synthesis [9]. At the molecular level, NP and AF cells display complementary expression patterns of lactate transport and metabolizing genes. NP cells predominantly express LDH-A (LDH5) and MCT4, facilitating lactate production and efflux, respectively. In contrast, AF cells are enriched in MCT1, LDH-B, and PDH, enabling them to take up and oxidize lactate efficiently [11]. This supports a model in which lactate produced by NP cells is exported via MCT4 and subsequently imported into AF cells through MCT1, where it is converted into pyruvate and enters the TCA cycle to fuel oxidative phosphorylation [9]. The schematic of lactate metabolism pathways is shown in Figure 1.

This inter-regional metabolic collaboration resembles the “lactate symbiosis” described in solid tumor microenvironments [81,82,83,84] and skeletal muscle [85], highlighting an adaptive strategy that enables the IVD to cope with nutrient limitations. It should be noted that a substantial portion of these findings was derived from disc cells cultured in monolayer, a condition known to induce phenotypic and metabolic shifts in cartilage-like cells, including enhanced mitochondrial content and oxidative metabolism [86].

**Lactate-mediated metabolic coupling between NP–EP.** The NP and CEP are anatomically and functionally integrated: the CEP provides mechanical support and governs solute transport, acting as the primary route for nutrient delivery and waste removal that defines the NP’s metabolic microenvironment and viability [87,88]. CEP stem cells promote NP regeneration and disc homeostasis, while NP-generated loads impose adaptive demands on CEP integrity—together establishing a bidirectional structural–functional coupling essential for disc health and for the disc’s response to degeneration [89,90,91].

Recent studies indicate that lactate functions as a “metabolic currency” among the NP, EP, and AF, sustaining motion-segment homeostasis through an MCT1-dependent, inter-tissue coupling. Specifically, recent in vivo studies suggest that during skeletal growth, glycolytic NP cells of the IVD exhibit elevated lactate secretion [80]; this lactate is taken up and reutilized by the adjacent EP (and, to a lesser extent, the AF) via MCT1, thereby supporting EP cartilage ossification and maturation. When MCT1-mediated lactate influx is impaired, a spectrum of phenotypes emerges—including reduced NP cellularity, exacerbated disc degeneration, and persistent, immature EP cartilage—underscoring the essential role of lactate coupling in disc development and health [80].

At the baseline metabolic level, both AF and EP display glycolytic features, yet their substrate preferences diverge in the presence of moderate glucose level (5 mM), EP cells efficiently utilize exogenous lactate, whereas AF cells, under the tested conditions, rely predominantly on glucose. ^13^C-lactate heavy isotope tracing experiment demonstrates that carbon from labeled lactate enters EP pyruvate and tricarboxylic-acid (TCA) intermediates and is incorporated into metabolites such as aspartate and malate, providing direct evidence for a “lactate → pyruvate → TCA” fuel pathway. By contrast, AF cells show markedly weaker lactate uptake and oxidation and instead favor glucose as the primary energy source—a pattern corroborated by Seahorse bioenergetics assays [80]. However, in low glucose (1 mM), AF cells readily utilize lactate as carbon source for energy production [9].

## 5. Lactate-Driven Epigenetic Regulation in the Intervertebral Disc

Lysine lactylation (Kla) is a relatively recently identified epigenetic modification, first reported by Zhang et al. in macrophages in 2019 [92]. This modification typically occurs at K14 and K18 on histone H3 and is strongly induced under high-lactate conditions such as hypoxia and infection. Similarly to classical lysine acetylation, histone lactylation is considered an activating mark that facilitates chromatin accessibility and transcription activation [92].

**Lactylation as a metabolic–epigenetic link in the IVD.** Lactylation is increasingly recognized as an important molecular mechanism linking metabolic status to epigenetic regulation within the intervertebral disc. In NP cells, lactate accumulation is closely associated with elevated levels of histone lysine lactylation (Kla), which contributes to the maintenance of disc homeostasis and the progression of intervertebral disc degeneration (IDD) by modulating chromatin structure and gene expression [93,94,95]. Multiple studies have demonstrated that, compared with healthy tissue, degenerated NP tissue exhibits significantly increased global lactylation (pan-Kla) levels, and that the proportion of Kla-positive cells rises with increasing degeneration severity, indicating a positive correlation between lactylation and IDD progression [95]. Further proteomic analyses have revealed that under hypoxic conditions—reflecting the physiological microenvironment of the disc—global protein lactylation is markedly upregulated in NP cells, with hypoxia-responsive Kla sites predominantly enriched in ribosomal and spliceosomal complexes as well as the VEGFA–VEGFR2 signaling pathway, suggesting potential roles for lactylation in protein translation, RNA splicing, and biological processes related to disc homeostasis [5].

Beyond the NP, lactylation in endplate cells has also been shown to be closely associated with metabolic status and transcriptional regulation. Lactate treatment significantly enhances global protein lactylation and histone H3K18 lactylation in endplate cells without affecting overall lysine acetylation levels, while studies using a conditional MCT1 knockout model demonstrate that restriction of lactate uptake in vivo is accompanied by a marked reduction in nuclear H3K18 lactylation in EP cells, indicating that this modification depends on intracellular lactate availability [80].

Building on these findings, potential therapeutic strategies targeting lactylation have begun to emerge. Shi et al. reported that a small-molecule compound targeting chromobox protein homolog 3 (CBX3) suppresses lactylation, restores extracellular matrix synthesis, and attenuates inflammatory matrix degradation, thereby alleviating IDD phenotypes [95]. In parallel, Zhang et al. identified a “glutamine–lactate–AMPKα lactylation axis” that enhances antioxidant capacity, autophagy, and resistance to senescence in NP cells [96]. Overall, these studies highlight lactylation as a central node in metabolic–epigenetic regulation within the intervertebral disc and underscore its potential as a diagnostic and therapeutic target for IDD.

**Mechanisms of Lactate–Acetylation Crosstalk in IVD Homeostasis and IDD.** Within the framework of metabolism–epigenetic crosstalk, a complex and dynamic interplay has been identified between lactylation and acetylation [92,97,98,99,100,101]. Both modifications utilize acyl-CoA intermediates derived from central carbon metabolism—lactyl-CoA and acetyl-CoA—reflecting the balance between glycolytic fermentation and oxidative phosphorylation [102,103]. Emerging evidence further indicates that Kla and lysine acetylation (Kac) can occur on identical histone residues (e.g., H3K18, H3K14), yet elicit distinct transcriptional outcomes through differential recruitment of reader proteins and competition for acyl-group donors [102,104]. In the hypoxic microenvironment of the IVD, the conversion of pyruvate to lactate is markedly enhanced. This metabolic shift reduces acetyl-CoA availability for acetylation while expanding the precursor pool for lactyl-CoA, thereby promoting lactylation [5,105,106]. Excessive lactate can also be converted into lactyl-CoA through the lactate dehydrogenase–acyltransferase system, further intensifying Kla [107].

At the enzymatic level, classical acetyltransferases such as p300/CBP have been shown to possess lactyltransferase activity under specific conditions. Likewise, histone deacetylases (HDACs) and sirtuins such as SIRT3 can catalyze both deacetylation and delactylation [108,109]. Consequently, Kla and Kac may compete or cooperate at the same or adjacent residues. For example, HDAC1/3 not only remove acetyl groups but also partially erase lactyl marks [100,110,111]. Interestingly, lactate has been shown to upregulate HDAC-related gene expression while paradoxically inhibiting HDAC enzymatic activity, ultimately promoting global histone hyperacetylation [106]. Hence, further research is needed to elucidate the interplay between Kla and Kac in IVD metabolic and epigenetic regulation.

## 6. Lactate-Mediated Cellular Signaling Pathways in the IVD

In addition to serving as a biofuel and substrate for histone lactylation, lactate through several recent studies also acts as a critical signaling molecule. In IVD cells, lactate—and the acidic microenvironment it generates—can activate multiple signaling pathways that regulate gene expression, cellular phenotype, and fate decisions. The following sections summarize the major signaling pathways through which lactate influences cellular behavior within the IVD.

**Dual Role of Lactate in Inflammation Regulation: From Pathological Stress to Immunometabolic Signaling.** In disc cells, Zhao et al. reported that lactate stimulation upregulates ASIC1a and ASIC3 in NP cells, increasing intracellular Ca^2+^ influx and ROS generation; this cascade activates NF-κB, promoting NLRP3 inflammasome assembly and release of IL-1β [72]. Furthermore, lactate can induce necroptosis, thereby exacerbate tissue damage and highlight its potential role as a danger-associated signal that facilitates disc degeneration under specific conditions [72].

In addition to experimental studies, a recent bioinformatics investigation by Sun et al. systematically analyzed intervertebral disc degeneration datasets and identified lactate metabolism-related genes that were closely associated with inflammatory signaling pathways and immune cell infiltration [112]. These findings further support the concept that altered lactate metabolism is linked to immunometabolic dysregulation during IDD progression, although functional validation is still required.

**Lactate-Related Akt Signaling and Cellular Senescence in the IVD.** High concentrations of lactic acid were shown to induce oxidative stress and cellular senescence in NP cells, effects that transcriptomic and bioinformatic analyses linked to activation of the PI3K/Akt signaling pathway [113]. Subsequent mechanistic studies confirmed that lactic acid interacts directly with Akt, modulating its downstream cascades—including Akt/p21/p27/cyclin D1 and Akt/Nrf2/HO-1 pathways—to promote oxidative stress and senescence in NP cells [113]. Molecular docking, site-directed mutagenesis, and microscale thermophoresis experiments further demonstrated that lactic acid binds to the Lys39 and Leu52 residues within the PH domain of Akt, thereby regulating its kinase activity [113].

**Lactate-Mediated Regulation of Ferroptosis: Signaling Pathways and Molecular Mechanisms in IVD.** Recent evidence suggests that ferroptosis also plays a pivotal role in IDD. In NP cells, elevated oxidative stress and lipid peroxidation can trigger ferroptosis, leading to cellular dysfunction, ECM degradation, and progressive structural deterioration of the IVD [3,114]. Xiang et al. [115] performed a comprehensive transcriptomic analysis of IDD tissues and controls using the Gene Expression Omnibus database and identified 80 ferroptosis-related differentially expressed genes (FRDEGs). Functional enrichment analysis indicated that these genes were mainly involved in responses to chemical stimuli and cellular stress, along with ferroptosis, TNF, HIF-1, NOD-like receptor, and IL-17 signaling pathways. Protein–protein interaction network analysis further identified 10 hub FRDEGs [115]. Receiver operating characteristic curve analysis based on the GSE124272 dataset demonstrated strong diagnostic potential of these core genes for IDD, and their expression trends were validated by RT-qPCR in a TBHP-induced NP cells degeneration model [115]. This study provided the first systematic characterization of ferroptosis-related genes in IDD and suggested potential molecular targets for early diagnosis and therapeutic intervention.

Several groups have further proposed ferroptosis-targeted therapeutic strategies by modulating iron homeostasis. Li et al. [116] reported that 1,25-dihydroxyvitamin D_3_ attenuated IDD progression by activating the vitamin D receptor pathway and suppressing ferroptosis in NP cells. Lu et al. [117] demonstrated that under oxidative stress, downregulation of the iron exporter ferroportin leads to intracellular iron accumulation and enhanced lipid peroxidation. Conversely, FPN overexpression upregulated glutathione peroxidase 4 (GPX4)—a key ferroptosis suppressor—reduced ROS levels, improved disc structure and function, and thereby delayed IDD progression [117].

**Figure 2:**
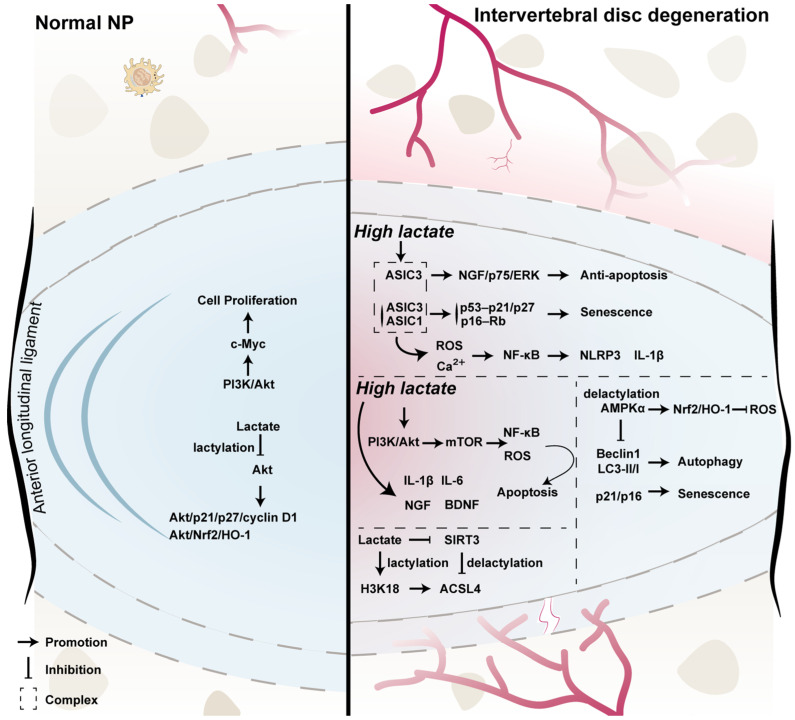
Lactate-mediated cellular signaling pathways in the IVD. The left panel depicts the physiological microenvironment of the NP, whereas the right panel illustrates the degenerative disc state. Under normal conditions, NP cells rely predominantly on glycolysis for energy production and continuously generate lactate. Within the hypoxic and avascular environment of the healthy disc, lactate, in addition to being a metabolic end product, may function as a signaling molecule by modulating PI3K/Akt–c-Myc signaling and lactylation-dependent regulation of cell proliferation, cell-cycle–associated molecules (e.g., p21, p27, and cyclin D1), and antioxidant responses (Akt/Nrf2/HO-1), thereby contributing to cellular homeostasis [96]. During intervertebral disc degeneration, abnormal accumulation of lactate in the local microenvironment is commonly accompanied by neovascularization, inflammation, and enhanced oxidative stress. Elevated lactate levels are proposed to activate acid-sensing ion channels (ASIC1/ASIC3), leading to Ca^2+^ influx and ROS generation, which subsequently activate NF-κB signaling and the NLRP3 inflammasome, promoting the expression of pro-inflammatory cytokines such as IL-1β [119]. Under acidic and high-lactate conditions, ASIC3 activation has been reported to upregulate nerve growth factor and activate ERK signaling via the p75 receptor, thereby enhancing NP cell tolerance to apoptotic stimuli under specific conditions; this pathway may be associated with neurovascular ingrowth and cell fate regulation in degenerative discs [119]. In parallel, lactate-associated signaling may engage classical cell-cycle inhibitory pathways, including p53–p21/p27 and p16–Rb, contributing to NP cell senescence, and may influence apoptosis, autophagy (Beclin-1, LC3-II/I), and oxidative stress through PI3K/Akt/mTOR and AMPKα-dependent signaling axes [36,113,120]. At the epigenetic level, lactate may promote histone lactylation (e.g., H3K18 lactylation), thereby modulating the expression of lipid metabolism–related genes, with ACSL4 proposed as a potential downstream target involved in lipid peroxidation and ferroptosis-related processes [118]. Conversely, the mitochondrial deacetylase SIRT3 participates in regulating the balance between lactylation and delactylation, thereby influencing mitochondrial function, oxidative stress, and cellular metabolic homeostasis [121,122]. It should be noted that many of the signaling pathways illustrated are derived primarily from in vitro studies and animal models, and their spatiotemporal relevance and relative contributions in the human intervertebral disc remain to be fully elucidated.

Recent studies demonstrate that lactate functions as a critical signaling molecule regulating ferroptosis through multiple mechanisms—particularly within the degenerative IVD microenvironment. Sun et al. [118] established a puncture-induced rat model of IDD and performed in vitro experiments with human NP cells to investigate the role of glycolysis-derived lactate in ferroptosis during disc degeneration. Multi-omics analyses revealed significantly elevated lactate levels during IDD, together with upregulation of acyl-CoA synthetase long-chain family member 4 (ACSL4), a key pro-ferroptotic enzyme. Mechanistically, lactate enhanced ACSL4 activity via two complementary pathways: (1) by inducing histone H3K18 lactylation, thereby promoting transcriptional activation of ACSL4, and (2) by inhibiting sirtuin 3 which increased ACSL4 protein lactylation and stability. These processes culminated in lipid peroxide accumulation and ferroptosis in NP cells [118]. In vivo, lentivirus-mediated overexpression of lactate oxidase significantly reduced lactate accumulation, suppressed ACSL4 expression and ferroptosis, and improved disc structure, thereby delaying degeneration. This study reveals that lactate contributes significantly to the progression of IVDD by triggering ferroptosis [118]. Lactate as a metabolic signal capable of modulating cell fate in IDD and propose potential targets for metabolic reprogramming–based therapies [118]. The schematic of lactate-mediated cellular signaling pathways in the IVD is shown in Figure 2.

## 7. Future Perspectives

Recent studies indicate that lactate in the IVD not only reflects cellular metabolic status but also participates in cell fate determination through coordinated signaling transduction and epigenetic regulation. Lactate-mediated signaling pathways intersect with histone and non-histone lactylation, collectively regulating inflammatory responses, cellular senescence, ferroptosis, and extracellular matrix homeostasis. However, how lactate integrates signaling and epigenetic mechanisms across distinct cell types and microenvironmental contexts, and how it exerts context-dependent effects during disc homeostasis and degeneration, remain to be systematically elucidated.

Meanwhile, accumulating evidence supports the existence of a lactate-centered metabolic coupling network among NP, AF, and EP cells, which may contribute to the coordinated maintenance of overall disc function. Nevertheless, the regulatory hierarchy, dynamic properties, and functional significance of this coupling network across different physiological and pathological stages remain poorly defined. In addition, inter-tissue lactate transport within the IVD warrants further investigation. Although the cartilaginous endplate and monocarboxylate transporters are recognized as key components of lactate exchange, how diffusion-based processes and transporter-mediated mechanisms cooperate under varying microenvironmental and mechanical conditions remains unclear.

## 8. Conclusions

There is a conceptual shift underway in our understanding of lactate, from being regarded merely as a ‘metabolic waste product’ to being recognized as a multifaceted metabolite with important roles in energy metabolism, signal transduction, and epigenetic regulation. These emerging functions are of particular relevance in IVD biology, given the intrinsically high-lactate microenvironment of the disc. Although the diverse roles of lactate in IVD homeostasis and degeneration are gaining increasing attention, the current body of evidence is derived predominantly from animal models and in vitro systems. Consequently, its context-dependent functions—especially those operating within the human intervertebral disc—remain to be rigorously validated.

Looking forward, the integration of multi-omics approaches, advanced molecular imaging technologies, and functional validation studies will be essential to systematically delineate the lactate-associated metabolic, signaling, and epigenetic networks within the IVD. Such efforts will provide a conceptual framework for understanding disc homeostasis and degeneration and will lay a foundation for the development of metabolism-based, disease-modifying therapeutic strategies.

## Data Availability

No new data were created or analyzed in this study.

## Abbreviations

**Abbreviation**	**Full Name**
ACAN	Aggrecan
AF	Annulus fibrosus
ASICs	Acid-sensing ion channels
ACSL4	Acyl-CoA synthetase long chain family member 4
BDNF	Brain-derived neurotrophic factor
CBX3	Chromobox protein homolog 3
CEP	Cartilage endplate
CEPCs	Cartilage endplate progenitor cells
CD147	Cluster of differentiation 147
CEST	Chemical exchange saturation transfer
c-Myc	Cellular myelocytomatosis oncogene
COL2A1	Collagen type II alpha 1
DCs	Dendritic cells
EAE	Experimental autoimmune encephalomyelitis
ECM	Extracellular matrix
ECAR	Extracellular acidification rate
EMMPRIN	Matrix metalloproteinase inducer
EPs	Endplate chondrocytes
ERK	Extracellular signal-regulated kinase
FOXO1	Forkhead Box Protein O1
FRDEGs	Ferroptosis-related differentially expressed genes
GLUT1	Glucose transporter 1
GLUT3	Glucose transporter 3
GPX4	Glutathione peroxidase 4
GAG	Glycosaminoglycan
GPR81	G protein–coupled receptor 81
GSK-3β	Glycogen synthase kinase-3β
G6P	Glucose-6-phosphate
H3K18	Histone H3 lysine 18
HDACs	Histone deacetylases
HIF	Hypoxia-inducible factor
HIF-1α	Hypoxia-Inducible Factor-1 alpha
IVD	Intervertebral disc
IDD	Intervertebral disc degeneration
IL-1β	Interleukin-1 beta
Kla	Lysine lactylation
Kac	Lysine acetylation
LBP	Low back pain
LDHA	Lactate dehydrogenase A
LDHB	Lacate dehydrogenase B
LDH5	Lactate dehydrogenase 5
MCT	Monocarboxylate transporter
MMP3	Matrix metalloproteinase 3
MMP2	Matrix metalloproteinase 2
MMP9	Matrix metalloproteinase 9
MMP13	Matrix metalloproteinase 13
NP	Nucleus pulposus
pan-Kla	Global lysine lactylation
PDK1	Pyruvate dehydrogenase kinase 1
PDH	Pyruvate dehydrogenase
ROS	Reactive oxygen species
SLC16A3	Solute Carrier Family 16 Member 3
TCA	Tricarboxylic acid
